# The Atomizer Sign: A Diagnostic Clue to Fragrance Allergic Contact Dermatitis

**DOI:** 10.5826/dpc.1103a41

**Published:** 2021-07-08

**Authors:** Eduardo Rozas-Muñoz, Jaime Piquero-Casals, Juan-Francisco Mir-Bonafé

**Affiliations:** 1 Department of Dermatology, Hospital San Pablo, Coquimbo, Chile; 2 Department of Dermatology, Clínica dermatológica multidisciplinar Dermik, Barcelona; 3 Department of Dermatology, Hospital Son Llàtzer, Palma de Mallorca, Spain

**Keywords:** Allergic contact dermatitis, fragrances, neck

## Case Report

**Figure 1:**
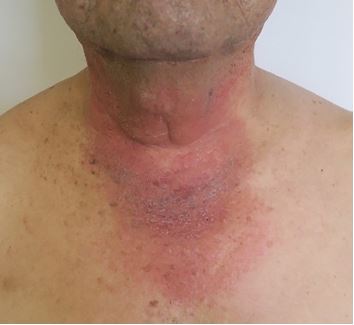
Eczematous plaques involving the anterior aspect of the neck (Adam’s apple region) and “V” of the anterior chest.

A 63-year-old man presented with pruritic and well-defined erythematous and vesicular eczematous plaques involving the anterior aspect of the neck and chest, present for the last 3 months (Figure 1). The rest of the physical examination was unremarkable, and there was no past or family history of seborrhea or psoriasis. The patient was treated with topical corticosteroids showing some improvements, but with frequent relapses. Patch test with Thin-Layer Rapid Use (T.R.U.E.) Epicutaneous Patch Test™ revealed a positive (+++) reaction to fragrance mix I. The patient was therefore instructed to avoid fragrance-containing products showing complete improvement of his dermatitis.

## Teaching Point

The presence of an eczematous dermatitis on the anterior neck in the Adam’s apple region has been referred to as the atomizer sign, being a common area of application of aerosol perfumes or cologne [1]. Patients with this peculiar presentation should be patch tested to rule-out any allergic contact dermatitis to fragrances.
